# Supplementary material for: Attention based GRU-LSTM for software defect prediction
Source: PLoS One. 2021 Mar 4;16(3):e0247444. doi: 10.1371/journal.pone.0247444 (PMC7932164; doi:10.1371/journal.pone.0247444)
Supplement: Attachment — Submitted filename: renamed_93a28.docx [file pone.0247444.s001.docx]

Dear Reviewer，

Thank you for your review of our manuscript (ID: PONE-D-20-33503), which is titled “**Attention Based GRU-LSTM for Software Defect Prediction**”. We appreciate your concerns and suggestions, and have revised our manuscript accordingly.

The followings are our responses for your comments.

Point 1:

Report all 10-fold experimental result for testing and learning.

**Response1:**

**Thank you for your** excellent suggestion. I have modified it according to your suggestion. **All the modifications are highlighted with Yellow in the paper.**

Point 2:

You only report results by considering radius equal to 4, but the proposed method can act differently with the radius equal to 2

**Response 2:**

**Thank you for your** excellent suggestion. I have modified it according to your suggestion. Table 5 and 6 inserted in the paper.

Point 3:

In “the three legends of accuracy, accuracy, and F measurement” you wrongly write “accuracy” instead of “precision”

**Response 3:**

**Thank you for your** excellent suggestions. **Yes, I have corrected it** “the three legends of Precision, Accuracy, and F-measurement”.

Point 4:

Explain more about figure 5 and 6.

**Response 4:**

**Thank you for your** excellent suggestion. We explained more after figures. **All the modifications are highlighted with Yellow in the paper.**

Point 5:

The only different exists between “SLDeep: Statement-level software defect prediction using deep-learning model on static code features” and your submission is in learning model, and DP-AGL model is based on SLDeep, DP-AGL is too similar to SLDeep. Is there any other novelty in your submission?

**Response 5:**

**Thank you for your** excellent suggestion. For this purpose, we made new section of “Discussion” also **highlighted with Yellow in the paper.**

Point 6:

Authors should upload the code associated to their published article so that readers can view and execute it (e.g. GitHub). Upload your code and link it to your article. It will allow users to re-run the analysis and reproduce the results.

**Response 6:**

**I have uploaded code but link is private until publish paper.** <https://github.com/shahbazshahbaz106/DP-AGL>

More reasons and modifications have been noted in the article, all the modifications are marked in Yellow in the paper. Special thanks to you for your good comments.

Sincerely yours,

Hafiz Shahbaz Munir, Shengbing Ren, Mubashar Mustafa, Chaudry Naeem Siddique, Shazib Qayyum,
